# APT weighted imaging in diffuse gliomas

**DOI:** 10.1259/bjro.20230025

**Published:** 2023-10-03

**Authors:** Lucia Nichelli, Moritz Zaiss, Stefano Casagranda

**Affiliations:** 1 Department of Neuroradiology, Sorbonne Université, AP-HP, Hôpitaux Universitaires La Pitié Salpêtrière - Charles Foix, Paris, France; 2 Department of Neuroradiology, University Clinic Erlangen, Friedrich-Alexander Universität Erlangen-Nürnberg (FAU), Erlangen, Germany; 3 Department of Research & Development Advanced Applications, Olea Medical, La Ciotat, France

## Abstract

Amide proton transfer-weighted (APTw) imaging is a non-invasive molecular MRI technique with a wide range of applications in neuroradiology and particularly neuro-oncology imaging. More than 15 years of pre-clinical experiments and clinical studies have demonstrated that APTw metrics are reproducible and reliable, leading to large-scale clinical acceptance. At present, major vendors of MRI scanners provide APTw sequences upon request. However, most neuroradiologists are unfamiliar with this advanced MRI contrast, its related metrics, and its established added value to patient care. In this manuscript, we present the APTw contrast and illustrate its clinical potential for glioma patients, before and after tumor therapy. We also show common artifacts of APTw imaging and discuss potential limitations and future refinements. Our goal is to suggest how this emerging technique can aid in diffuse gliomas work-up.

## Introduction: the novelty of APTw contrast

Amide proton transfer-weighted (APTw) imaging is an MRI technique based on the principles of chemical exchange saturation transfer (CEST). In CEST imaging, molecules in the millimolar range with exchangeable protons are detected indirectly by measuring the intensity changes of water protons before and after the application of a radiofrequency (RF) pulse train. The RF pulse train is selectively applied to the resonance frequency of the proton group of interest leading to a depletion of the magnetization of these protons, also called saturation. This saturation is transferred *via* chemical exchange to non-excited water protons. During the saturation period, proton exchange is continuously repeated to enhance the detectability of low-concentration molecules. In APTw imaging, the chemical species of interest are amide protons of mobile peptides and proteins: thus, the effect detected by water signal intensity larger when the protein concentration is higher.

Tumors have an increased protein content compared to normal brain tissue,^1^ partially explaining their APTw hyperintensity pletely non-invasive.^2,3^ This contrast mechanism provides information at the subcellular protein level, justifying the term “molecular” technique, and does not require an exogenous contrast agent, so it is completely non-invasive. It should be noted that amide protons are not the only contributors to APTw contrast, but several confounding effects compete with saturation transfer (such as Nuclear Overhauser Enhancement, Magnetization Transfer and Direct water Saturation), and disentangling their contributions is complex. Therefore, the term “weighted” is preferred to the solely “APT imaging” and APTw acquisition protocols should strictly follow expert recommendations for APTw brain tumor imaging at 3 T^2^ to avoid improper qualitative or quantitative evaluation of APTw maps.

**Figure 1.**
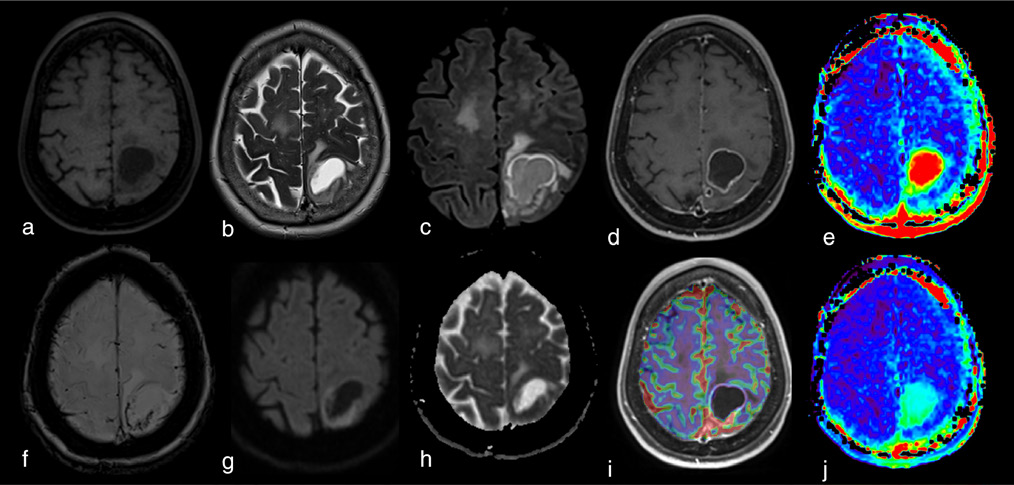
Example of a high-grade glioma (Glioblastoma, IDH-wildtype, WHO grade 4) studied with an MRI multimodal imaging protocol that includes APTw imaging before (e) and after (j) fluid suppression. Notice how the cystic component of the tumor has a high signal intensity (red) in E and lower signal intensity (green) after the fluid suppression. In contrast, tissular components in the posterior part of the lesion maintain their hyperintensity after fluid suppression, suggesting malignancy. (a) three-dimensional T1-weighted spin echo sequence (3D T1w SE). (b)T2 weighted (T2w) image. (c) 3D Fluid attenuated inversion recovery (FLAIR) weighted sequence in the axial plane. (d) 3D T1w SE sequence in the axial plane after contrast injection (3D T1w SE injected). (e) APTw map. (f) susceptibility weighted imaging (SWI). (g) axial diffusion weighted imaging (DWI), b value of 1000 s/mm^2^. H: Apparent Diffusion Coefficient (ADC) map. I: relative Cerebral Brain Volume (rCBV) map after leakage correction superposed to 3D T1w SE injected sequence. J: Fluid-suppressed APTw image (F.S. APTw). All APTw images displayed in this manuscript were acquired at 3 T Skyra or Prisma scanner (Siemens Healthineers, Erlangen, Germany) with a 3D snapshot-GRE sequence^5^ using a B1 root-mean square value of 2 µT. B0 artifact were corrected with WASAB1 protocol. If not otherwise specified, the duty–cycle was of > 90%. Olea Sphere 3.0 software (Olea Medical, La Ciotat, France) was used to denoise APTw data, to correct them from motion, to normalized them to M0, for B0 correction, and to compute APTw and F.S. APTw maps respectively based on the MTRasym^2^ and MTRRex.^6^ APTw maps were visualized with a RAINBOW palette between -1% and 5%. ADC, apparent diffusion coefficient; APTw, amide proton transfer-weighted; CEST, chemical exchange saturation transfer; FLAIR, fluid attenuated inversion recovery; rCBV, relative cerebral brain volume.

Recent consensus guidelines for APTw neuro-oncology imaging explain the choice of pulse sequences, acquisition protocols, and data processing methods at 3 T.^2^ Concerning data processing approaches, magnetization transfer ratio asymmetry relative to the water frequency (MTRasym) is the recommended metric to compute APTw contrast, even though alternative methods have been proposed.^4^ MTRasym can be complemented by fluid-suppressed metrics to improve image readability^2^ (Figure 1).

To conclude, APTw is a molecular MRI technique that non-invasively provides tissue information at the protein level due to complex synergistic processes. Recent consensus guidelines for APTw imaging of brain tumors will hopefully harmonize data acquisition and interpretation and may facilitate APTw implementation in clinical protocols. We will now review how can APTw contrast can impact on glioma management.

## APTw findings in untreated gliomas

**Figure 2.**
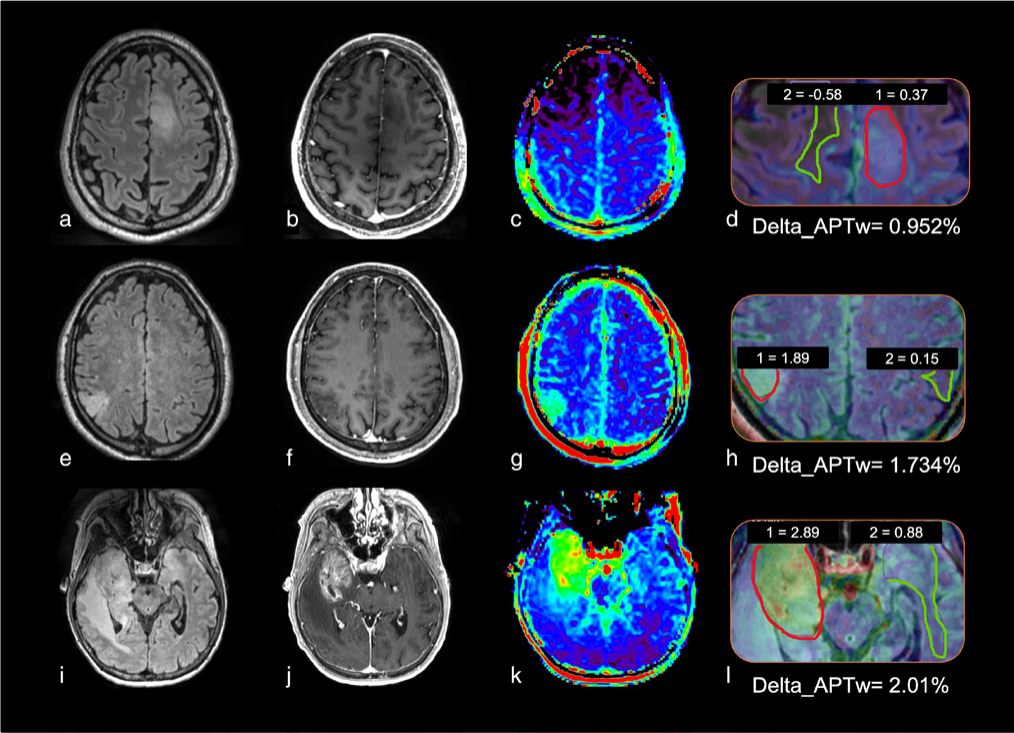
Example of how APTw signal intensity increases as a function of tumor grade *(case courtesy of Sotirios Bisdas and Laura Mancini, University College London)*. (a–d) Oligodendroglioma, IDH-mutant and 1p/19q codeleted, WHO grade 2. (e–h) Oligodendroglioma, IDH-mutant and 1p/19q codeleted, WHO grade 3. (i–l) glioblastoma, IDH-wildtype, WHO grade 4. Lesion measurements analysis is displayed in D, H and L: average values of APTw metrics are computed after delineation of a region of interest (ROI) in the tumor lesion and in the contralateral normal-appearing white matter (cNAWM). Delta_APTw is calculated after the subtraction of the APTw ROI lesion values with the cNAWM ROI values. (a, e, l) FLAIR sequence. (b, f, j) T1w after injection. (c, g, k) APTw maps. (d, h, l) zoom on APTw maps superposed with FLAIR images and Delta_APTw measurements. APTw, amide proton transfer-weighted; cNAWM, contralateral normal-appearing white matter; FLAIR, fluid attenuated inversion recovery; ROI, region of interest.

APTw signal intensity increases with increasing histological grade of diffuse gliomas.^7,8^ Therefore, APTw contrast is lower in low-grade gliomas (LGGs) and higher in high-grade gliomas (HGGs), and it also differs in Grade 2 from Grade 3 (Figure 2). If a standardized protocol is used, APTw signal intensity doesn’t significantly change between different centers or vendors and ranges from 1 to 4% in Grade 2 to 4 diffuse gliomas.^2^

**Figure 3.**
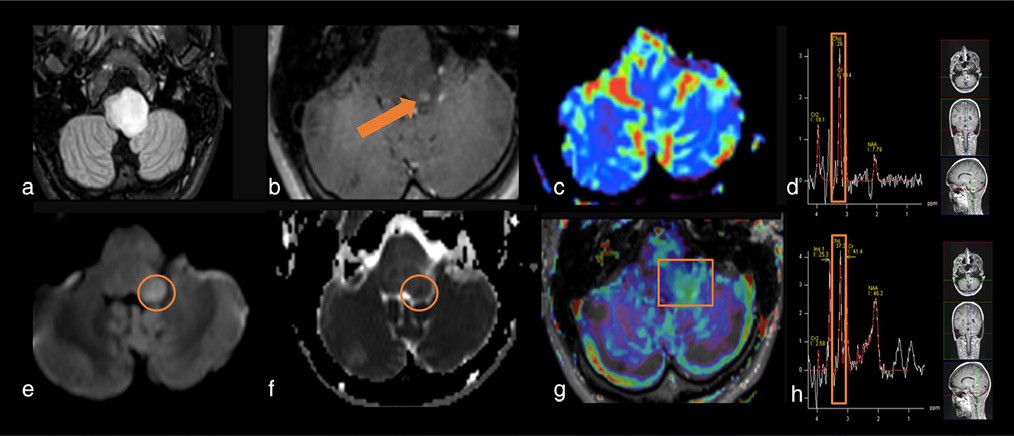
Diffuse midline glioma, H3 K27- altered (WHO grade 4), pre-operatively studied with a multi-modal protocol that included APTw sequence. Notice that the APTw hyperintensity of the left posterior part of the bulbar lesion (orange square) correspond to the area of tumor restriction (orange circle) and is related to an increased choline peak in MRS (orange rectangle). Interestingly, the area of APTw increased signal intensity goes beyond contrast enhancing area (arrow) and is associated with a weak hyperperfusion (C). (a) Axial 3D FLAIR sequence. (b) Axial 3D T1w after contrast injection. (c) rCBV map, after leakage correction. (d) long echo time spectrum centered on the FLAIR lesion. (e) axial diffusion tensor imaging (DTI), 12 directions. (f) ADC derived cartography. (g) F.S. APTw map, superposed to T1w injected sequence. For this case, a 55% duty cycle protocol was used. (h) short echo time spectrum centered on lesion visible on FLAIR. ADC, apparent diffusion coefficient; APTw, amide proton transfer-weighted; FLAIR, fluid attenuated inversion recovery; rCBV, relative cerebral brain volume.

These findings can possibly be explained by the increase in cellularity, and ultimately in protein content, in higher grades.^3^ The hypothesis is supported by the positive correlation between histological markers (cell density, cell count, mitotic index) and APTw signal intensity.^8^ In addition, APTw signal intensity correlates with other MRI tumor proliferation and density markers, as choline/creatine ratio in magnetic resonance spectroscopy (MRS), and apparent diffusion coefficient (ADC) in diffusion-weighted imaging (DWI) (Figure 3).

When compared to four advanced DWI models, APTw imaging shows increased diagnostic accuracy.^9^

**Figure 4.**
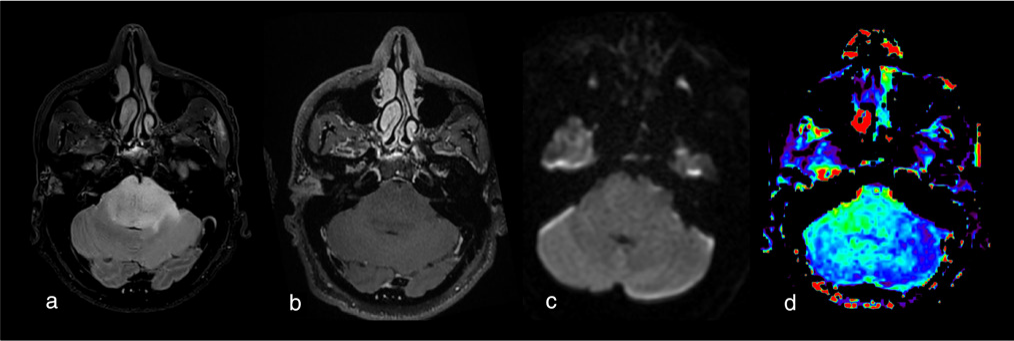
Probable diffuse brainstem glioma (no histology available) displaying high APTw signal intensity value within the lesion (especially in its right part) and therefore suggesting neoplastic malignancy. Of note, no tumor enhancement (in B) nor diffusion restriction (C) or neoangiogenesis (image not shown) is seen within the lesion. (a) 3D FLAIR. (b) 3D T1w after contrast injection. (c) DWI. (d) F.S. APTw map. For this case, a 55% duty cycle protocol was used. APTw, amide proton transfer-weighted; FLAIR, fluid attenuated inversion recovery.

**Figure 5.**
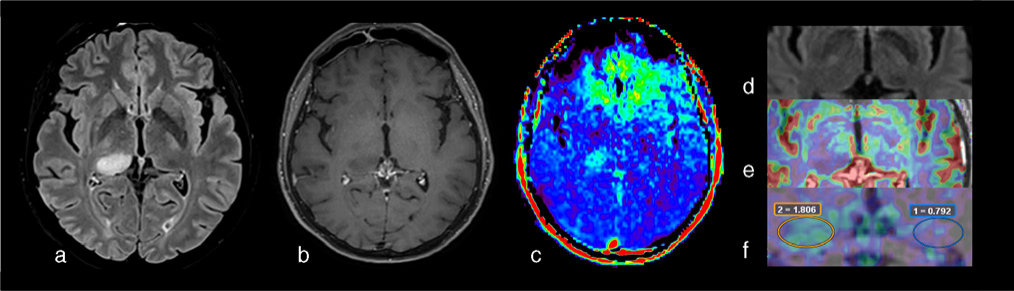
Example of a molecular defined glioblastoma (astrocytoma with *EGFR* gene amplification, therefore glioblastoma IDH-wildtype, WHO grade 4) displaying a well-limited lesion without mass effect, contrast enhancement, diffusion restriction nor hyperperfusion but with high APTw values. (a) 3D FLAIR. (b) 3D T1 w after injection. (c) APTw map. (d) axial diffusion, b value of 1000 s/mm^2^. (e) rCBV map after leakage correction superposed to T1w contrast injected sequence. (f) APTw map superposed to 3D T1w injected sequence. APTw, amide proton transfer-weighted; FLAIR, fluid attenuated inversion recovery; rCBV, relative cerebral brain volume.

Current advanced MRI protocols cannot replace the full relevant prognostic information provided by biomolecular analysis on surgical tissue samples. Nevertheless, preliminary non-invasive information can be very helpful for promptly orienting therapeutic strategies. For example, a lesion may be surgical inaccessible (Figure 4), patients may not be able to tolerate or may refuse a surgical procedure, biopsy samples may be insufficient or damaged, and some tumor entities may require additional molecular testing that delay proper immediate patient care. In these contexts, MRI molecular information can significantly improve patient management. Moreover, HGGs can manifest as a non-enhancing tumor and thus mimic a lower-grade neoplasm (Figure 5). Adding APTw imaging to these patients may improves MRI’s diagnostic accuracy, as it can possibly aid in the prognostic stratification of non-enhancing gliomas.^3^

Important diagnostic and prognostic biomarkers have been shown to correlate with APTw contrast, most notably IDH mutation and MGMT methylation status. Therefore, APTw may, highlight different subtypes of underlying tumor malignancy.^10,11^

**Figure 6.**
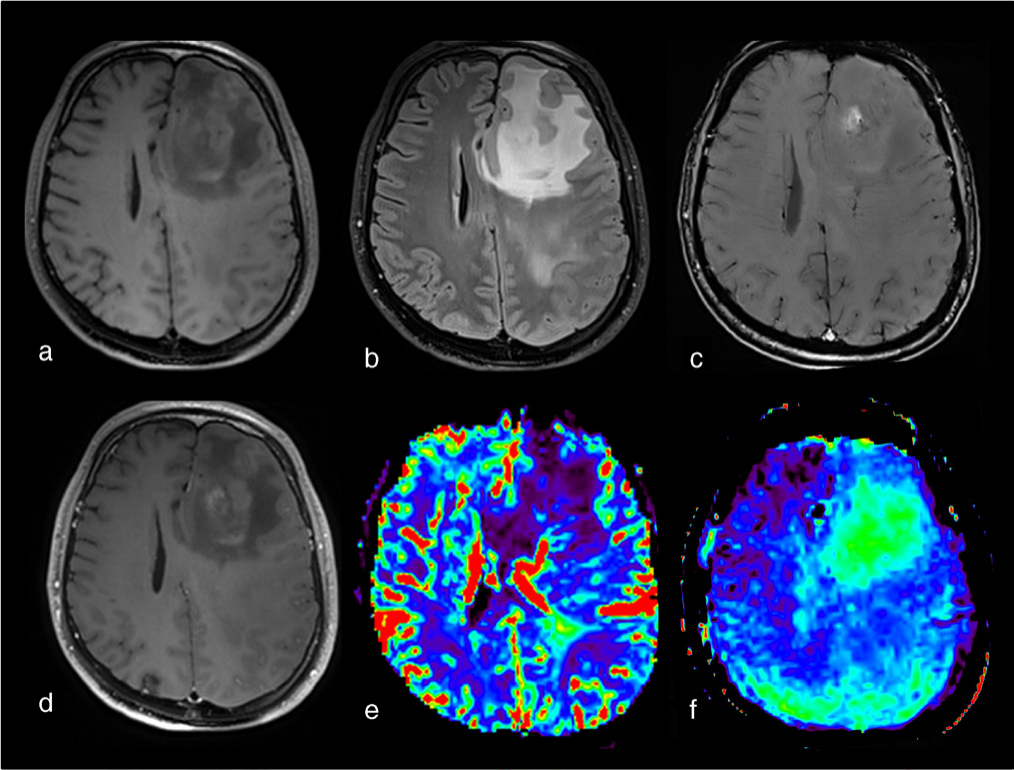
Another example of a non-treated glioblastoma (DH-wildtype, WHO grade 4) that displays high malignant features on the APTw sequence in contrast to DSC perfusion. (a) 3D T1w SE in the axial plane showing an intra-axial lesion with mass effect on brain parenchyma and lateral ventricles. (b) 3D FLAIR. (c) SWI sequence showing some hemorrhagic components. (d) 3D T1w SE injected sequence showing mild homogenous contrast enhancement. (e) rCBV map after leakage correction. (f) A F.S. APTw map. For this case, a 55% duty cycle protocol was used. In the posterior part of APTw image B0 artifact are seen. 3D, three-dimensional; APTw, amide proton transfer-weighted; cNAWM, contralateral normal-appearing white matter; FLAIR, fluid attenuated inversion recovery; ROI, region of interest.

Indeed, a fast-growing body of evidence highlights the prognostic information provided by APTw contrast in brain gliomas.^12,13^ In the modern era of neuro-oncology, accurate stratification of patient outcome is essential to tailor treatment strategy and therefore prediction of patient survival is of strong clinical relevance^13,14^ (Figures 5 and 6).

**Figure 7.**
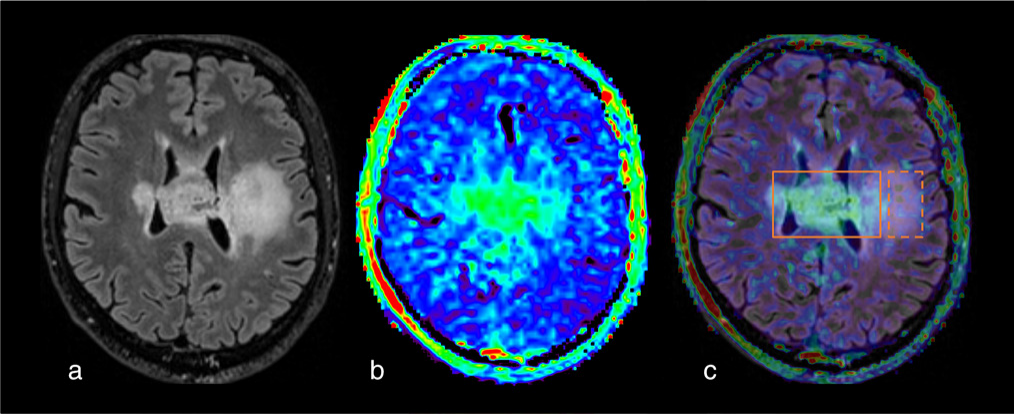
Example of a relatively homogenous FLAIR signal intensity and heterogenous APTw contrast, that might reflect the ability of APTw imaging to distinguish between predominantly neoplastic infiltration (orange rectangle, higher APTw signal intensity values) and vasogenic edema (smaller dotted rectangle, lower APTw signal intensity values) in a treated glioblastoma (IDH-wildtype, WHO grade 4) (*case courtesy of Sotirios Bisdas and Laura Mancini, University College London*). (a) 3D FLAIR. (b) APTw map. (c) F.S. APTw map superposed to FLAIR sequence. Of note, no histological examination or follow-up information can confirm the presence of tumor infiltration and/or vasogenic edema in this patient. APTw, amide proton transfer-weighted; FLAIR, fluid attenuated inversion recovery.

**Figure 8.**
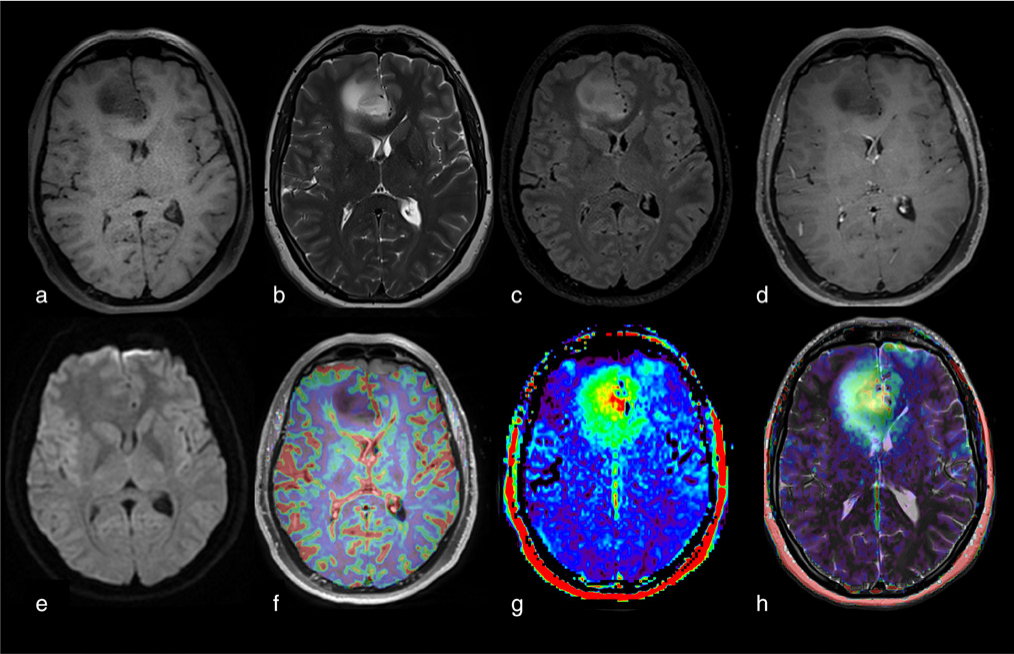
Example of possible (but not histologically proved) tumoral infiltration visible with APTw imaging and not with morphological MRI sequences in a pre-treated astrocytoma (IDH-mutant, WHO grade 4). Notice the high APTw signal intensity in the genu of the corpus callosum (orange rectangle) without T2-FLAIR hyperintensity, hypercellularity, or neo-angiogenesis. No histological examination or follow-up information can affirm the presence of tumor infiltration beyond the FLAIR signal. (a) T1w sequence. (b) T2w. (c) FLAIR. (d) T1w injected sequence. (e) DWI. (f) rCBV map after leakage correction superposed to T1w injected sequence. (g) APTw map. (h) F.S. APTw map superposed to T2w sequence. APTw, amide proton transfer-weighted; FLAIR, fluid attenuated inversion recovery.

In addition to the reliable value of APTw quantitative metrics, the visual distribution of APTw signal intensity in APTw maps could provide pivotal spatial information. For example, APTw cartography seem to depict glioma heterogeneity and therefore may target tissue biopsies to reduce sampling errors and underestimation of tumor grade. It can also portray the concentration of malignant tumor cell within a non-enhancing peritumoral signal abnormality,^3^ thus possibly disentangling between vasogenic edema and neoplastic infiltration (Figure 7) and could help in estimating tumor boundaries (Figure 8). More accurate visualization of glioma boundaries has a major potential impact on glioma patient care, as it may help increase the rate of complete surgical resection or improve target volume delineation for radiotherapy.

**Figure 9.**
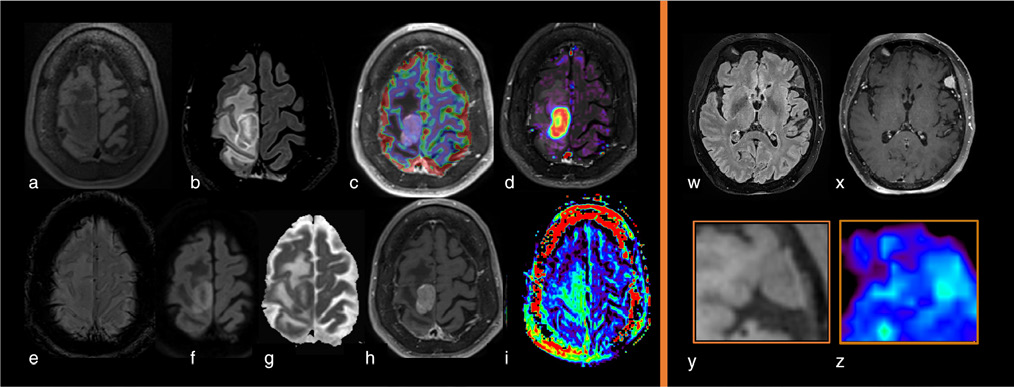
Example of a primary central nervous system lymphoma (A – I) and of a meningioma (W – Z), both displaying high signal intensity on F.S. APTw imaging (see I for lymphoma and Z for meningioma). (a) 3D T1w SE. (b) 3D FLAIR. (c) rCBV map after leakage correction superposed to T1w injected sequence. (d) K2 cartography (derived from DSC perfusion) superposed to FLAIR. (e) SWI. (f) DWI. (g) ADC map. (h) 3D T1w injected sequence. (i) APTw map. Of note, APTw hyperintensity goes beyond contrast enhancement and covers the quasi-totality of the FLAIR contrast. (w) 3D FLAIR. (x) axial 3D T1w injected sequence. (y) zoom on the extra-axial lesion on T1w without contrast injection. (z) zoom on APTw map. 3D, three-dimensional; ADC, apparent diffusion coefficient; APTw, amide proton transfer-weighted; FLAIR, fluid attenuated inversion recovery; rCBV, relative cerebral brain volume.

**Figure 10.**
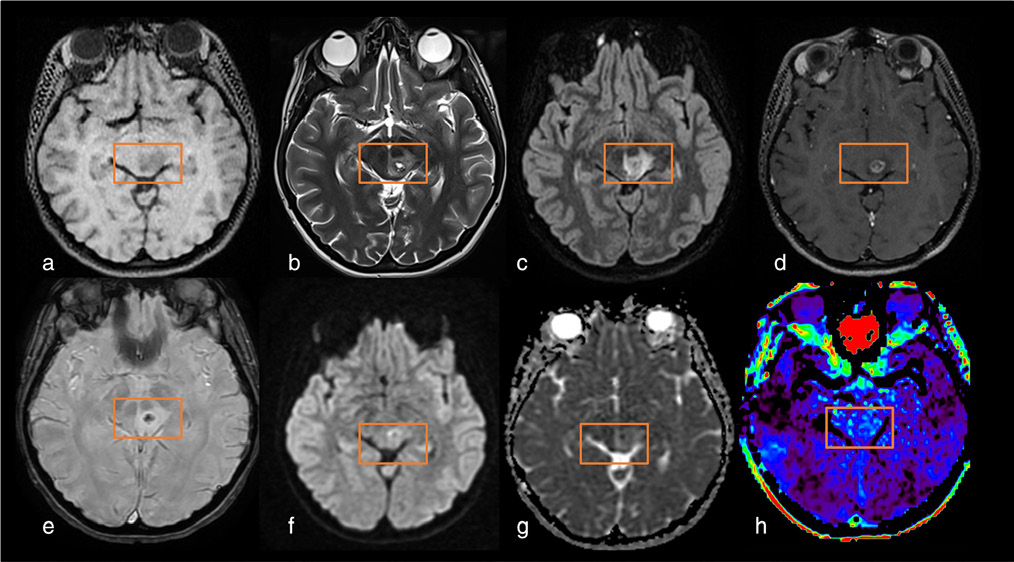
Example of a Neuro-Behçet disease studied before biopsy with a multimodal protocol that included APTw imaging (H). Histological samples showed necrotic and inflammatory non-specific features without infectious agents. Clinically, diagnostic criteria for Behçet's disease were fulfilled. The lesion was effectively treated with corticosteroids. A: 3D T1 SE. B: T2w. C: 3D FLAIR. D: 3D T1w injected sequence. E: SWI. F: DWI. G: ADC map. H: F.S. APTw image showing no increased APTw signal in the left mesencephalic necrotic-hemorrhagic lesion. 3D, three-dimensional; ADC, apparent diffusion coefficient; APTw, amide proton transfer-weighted; FLAIR, fluid attenuated inversion recovery; rCBV, relative cerebral brain volume.

Lastly, APTw imaging has been extensively studied in gliomas, but limited literature exists concerning APTw intensity values of other tumoral and non-tumoral brain lesions. Meningiomas and lymphomas show elevated APTw metrics (Figure 9), and demyelination is also known to increase APTw contrast.^3^ To date, there are no cut-off values to distinguish these entities. Therefore, caution must be taken when interpreting APTw imaging in a pre-surgical evaluation of a brain mass, and the final MRI diagnosis should incorporate qualitative and quantitative data from the whole conventional and advance MRI protocol (Figure 1). At the same time, in some cases APTw imaging can aid on orienting toward a non-tumoral nature of a lesion (Figure 10), but still little is known about APTw predictive values in the characterization of a brain space-occupying lesion.

To summarize, APTw imaging increases the current diagnostic accuracy of MRI in predicting glioma grade, molecular markers and prognosis. Moreover, APTw contrast may allow the visualization of glioma heterogeneity and tumor infiltration.

## APTw findings in treated gliomas

**Figure 11.**
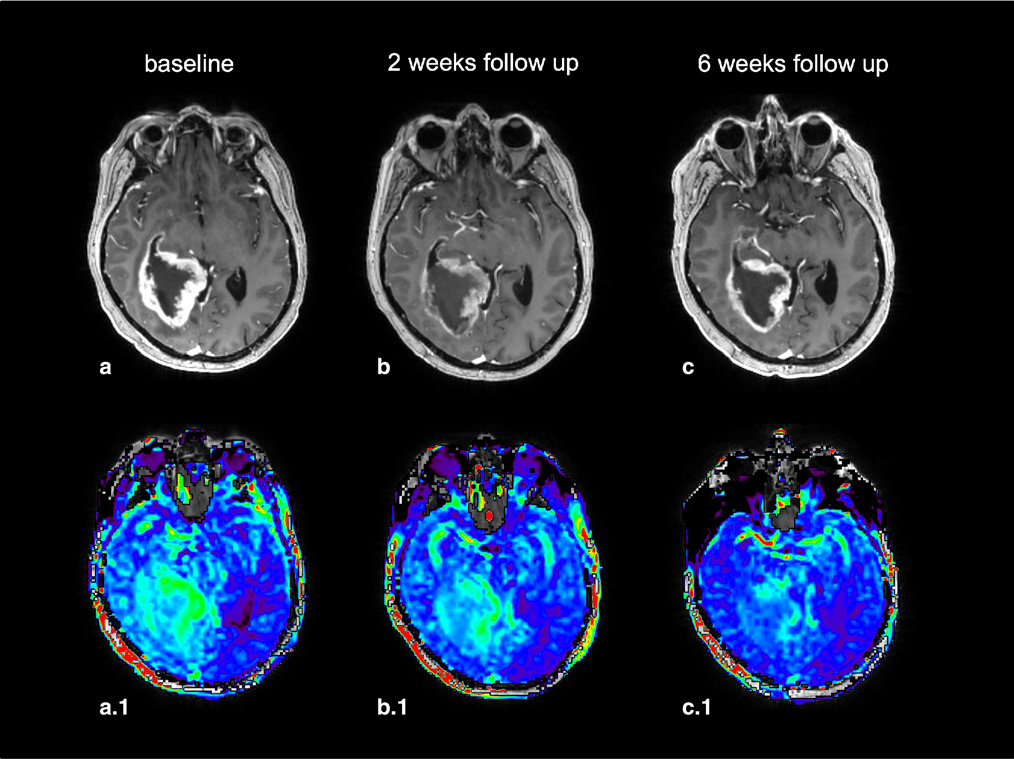
Example of a glioblastoma (IDH-wildtype, WHO grade 4) before STUPP protocol (radiotherapy and concomitant chemotherapy with temozolomide, a and a.1), during STUPP protocol (b and b.1) and at the very end (c and c.1) (*case courtesy of Sotirios Bisdas and Laura Mancini, University College London*). (a, b, c) 3D T1w injected sequence, showing no significant tumor volume decrease (stable disease according to RANO criteria). (a.1, b.1, c.1) F.S. APTw map, showing a rapid (b.1) and important decrease of APTw signal intensity, notably in the left and in the posterior tissular part of the lesion. APTw, amide proton transfer-weighted.

To date, APTw imaging in treated gliomas has been less explored than in the untreated group, but results are very encouraging^15–17^ (Figure 11). The clinical added value of a reliable non-invasive molecular approach in a follow-up setting is clear, since MRI is essential to determine response to treatment and since diagnostic uncertainties between tumor recurrence and post-therapeutic changes are very common. In these contexts, repeating surgical procedures is not feasible and not having a diagnosis, and therefore delaying therapeutic decisions, is undesirable.

**Figure 12.**
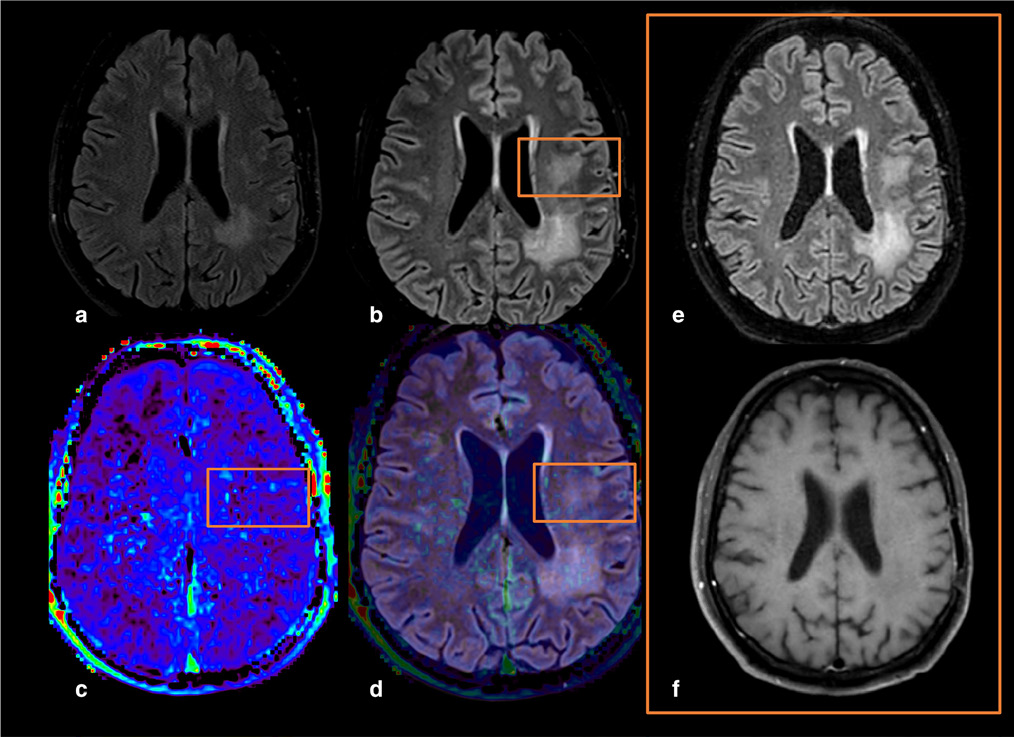
Example of a glioblastoma (IDH-wildtype, WHO grade 4) five months (a) and seven months after STUPP protocol (b), showing the appearance of a non-contrast FLAIR hyperintensity in the frontal left corona radiata (little orange rectangle) that didn’t show any increase in APTw signal intensity, therefore suggesting a non-tumoral, post-therapeutic (probable radiation-induced leukoencephalopathy) lesion, as confirmed by follow-up examination (e-f). (a,b) 3D FLAIR images at different time points (five and seven months after STUPP). (c) F.S. APTw image. (d) F.S. APTw map, superposed to FLAIR image, (e,f) two-months follow-up after B, with 3D FLAIR (e) and 3D T1w after injection (f) that confirm the lesion stability with no contrast enhancement, and therefore strongly suggest then non-tumoral nature of the lesion. 3D, three-dimensional; ADC, apparent diffusion coefficient; APTw, amide proton transfer-weighted; FLAIR, fluid attenuated inversion recovery.

APTw signal intensity is increased in recurrent tumor lesions and shows lower values in pseudo-progression and in radio-necrosis, reflecting low or absent cellularity in post-therapeutic remnants.^17^ In addition, APTw contrast is predictive of early progression after treatment^18^ and can assist in the evaluation of non-enhancing peritumoral areas, especially as they appear and/or progress (*e.g.* after radiotherapy, as in Figure 12).

Compared to multiparametric MRI protocols that include DWI and MRS, APTw imaging has higher diagnostic performance in the distinction between glioma progression and treatment effects.^19^ Compared to methionine positron emission tomography (MET-PET), it also appears to provide a more accurate diagnosis.^20^ Additionally, a voxel-wise analysis between APTw maps and MET-PET shows a significant correlation in LGG but not in HGG, suggesting that even though these techniques are both a surrogate for protein metabolism, they reflect different biophysical processes.

It is also of note that APTw imaging seems to improve therapeutic assessment compared to conventional MRI sequences,^15,16^ as it enables earlier distinction between responders and non-responders patients (Figure 11). Other advanced MRI tools, especially perfusion-weighted imaging and MRS, seem to provide complementary information to APTw contrast. Further histologically validated studies need to unravel their optimal contribution to clinical practice.

## Technical considerations, pitfalls and future perspectives

**Figure 13.**
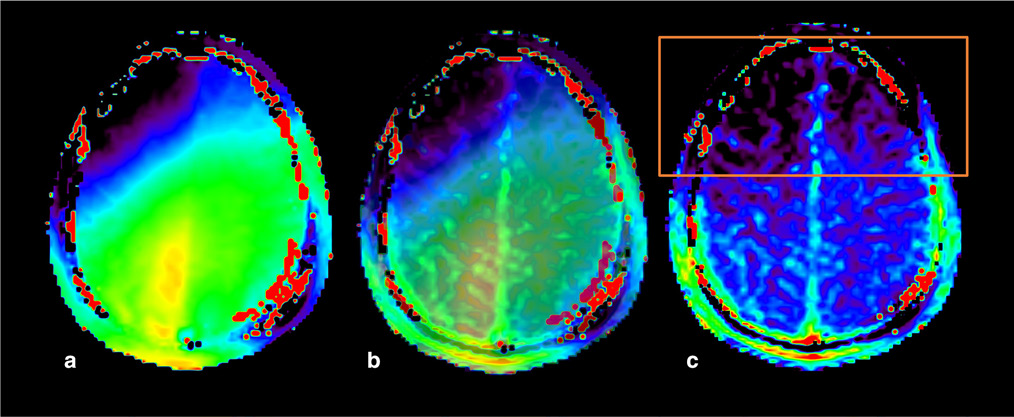
Example of B1 field inhomogeneities artifact in a healthy volunteer (*case courtesy of Sotirios Bisdas and Laura Mancini, University College London*). Relative B1 map (a), APTw map (c) and their overlay (b). B1 artifact on the APTw map is responsible for anterior frontal bilateral hypointensities.

**Figure 14.**
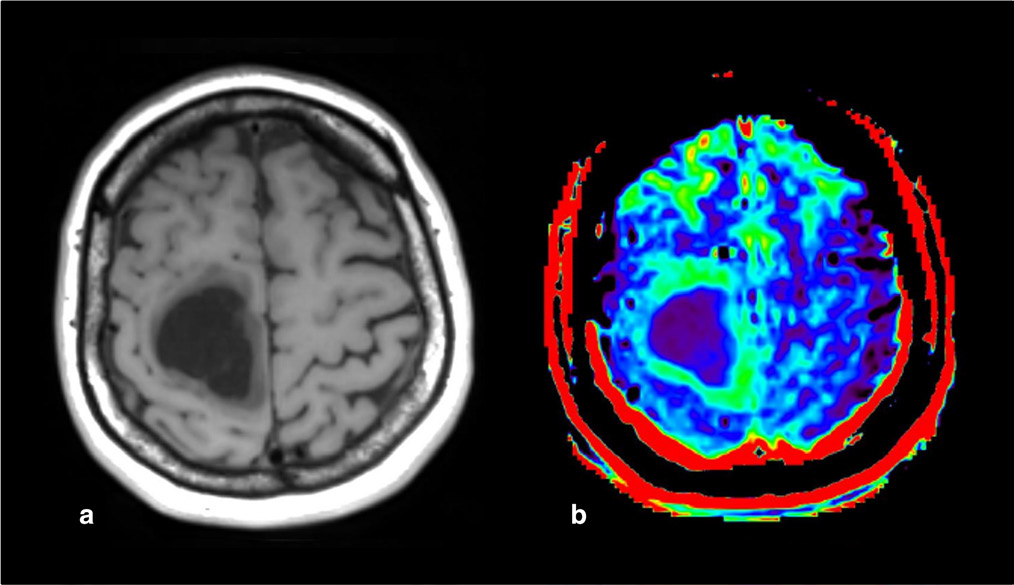
Example of inadequate lipid suppression in a glioblastoma (IDH-wildtype, grade 4) (*case courtesy of Sotirios Bisdas and Laura Mancini, University College London*). (a) 3D T1w SE sequence and (b) APTw map. No lipid suppression method was used, leading to a strong hyperintensity in the skull. For this case, a 2-D EPI 53% duty cycle protocol was used. 2D, two-dimensional; APTw, amide proton transfer-weighted.

**Figure 15.**
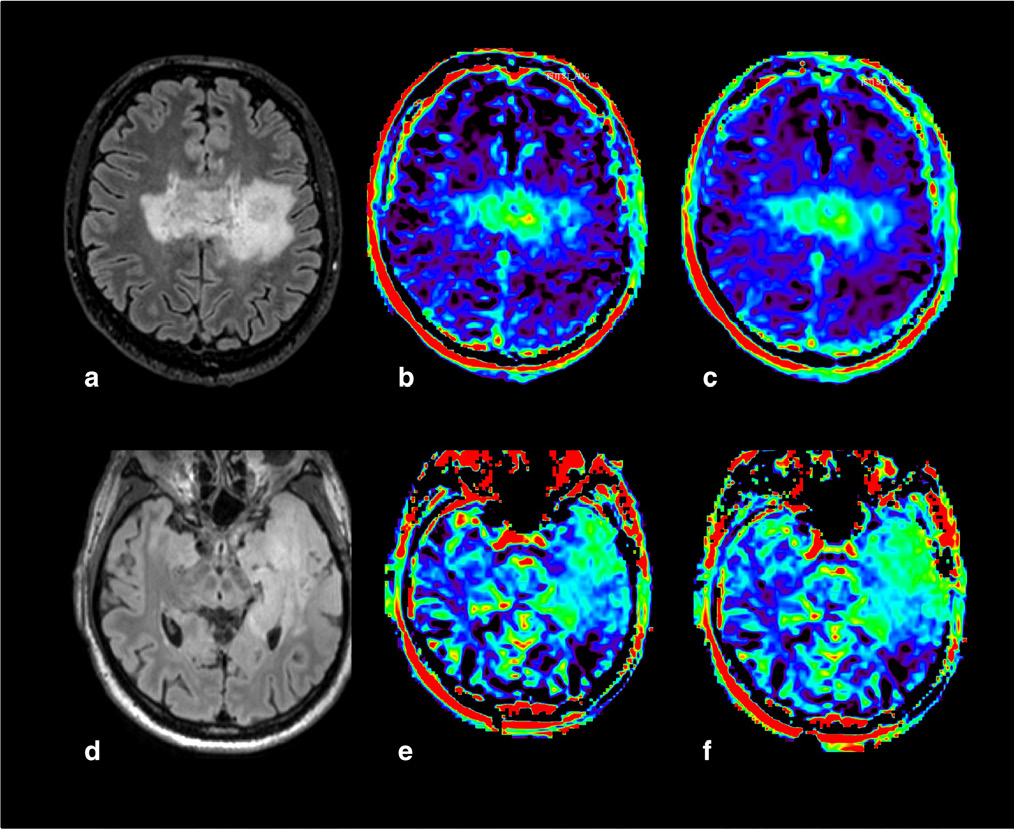
Example of motion artifacts in two glioblastomas (IDH-wildtype, grade 4) (*case courtesy of Sotirios Bisdas and Laura Mancini, University College London*). (a) 3D FLAIR of the case depicted on Figure 7. (b) APTw map before motion artifacts correction. (c) APTw map after an adequate motion artifact correction. (d) 3D FLAIR. (e) APTw map before motion artifacts correction. (f) APTw map after an inadequate motion artifact correction due to too strong kinetic movements. 3D, three-dimensional; ADC, apparent diffusion coefficient; APTw, amide proton transfer-weighted; FLAIR, fluid attenuated inversion recovery.

Like any MRI technique, APTw imaging displays artifacts whose contributors should be considered when interpreting APTw maps. Image quality can be damaged mainly from B_0_ and B_1_ field inhomogeneities (Figure 13), inadequate lipid suppression (Figure 14) and from motion (Figure 15). Of note, it is recommended to perform APTw imaging before gadolinium injection to avoid APTw signal bias.

Lipid artifacts occur primarily in the skull and should be removed with a standard spectral pre-saturation inversion-recovery method.

B_0_ field inhomogeneities are prominent near air–tissue interfaces, as in mastoids or paranasal sinus, and can be adjusted if a B_0_ inhomogeneity map (ΔB_0_ map) of the same geometry is acquired, allowing to realign water center frequency.^2^ A ΔB_0_ map is usually implemented in the APTw protocol as a separate acquisition and B_0_-corrected APTw maps are usually provided by vendors. Advanced post-processing approaches should also correct inhomogeneities, motion, noise and, importantly, artifacts due to fluid components. Indeed, large vessels, cystic or hemorrhagic areas and liquefactive necrosis demonstrate high APTw signal intensities. The mechanism beyond these hyperintensity artifacts is complex, and mostly due to spillover dilution effects^5,6^ that dilute CEST effects in solid tissue, but to a lesser amount in more fluid environments. In the future, the validation of fluid-suppressed metrics is advisable, as it will allow more accurate pre- and post-therapeutic tumoral assessment.

The delineation of the region of interest in a tumor and its normalization with the contralateral normal-appearing white matter (Figure 2) should also be standardized and hopefully automatized to reduce measurements variations.

Finally, the anterior frontal lobe is more sensible to B_1_ inhomogeneities (Figure 13), while the posterior fossa is more sensible to both B_0_ and B_1_ inhomogeneities, affecting APTw signal repeatability. Currently, no robust B_1_ correction approach has been proposed for APTw imaging. Therefore, caution should be taken when analyzing anterior frontal lobe and infratentorial lesions.^2^

## Conclusion

APTw imaging may allow to improves glioma patient care and might offer complementary information to conventional and advanced MRI approaches. Attention should be paid to adjusting APTw parameters to the recently standardized guidelines, in order to correctly interpret APTw signal intensity. In the future, emerging techniques for fluid suppression will increase APTw accuracy and furtherly refine molecular correlations.
